# Toxic Elements in Different Medicinal Plants and the Impact on Human Health

**DOI:** 10.3390/ijerph14101209

**Published:** 2017-10-11

**Authors:** Eid I. Brima

**Affiliations:** Department of Chemistry, Faculty of Science, King Khalid University, P.O. Box 9004, Abha 61413, Saudi Arabia; ebrahim65@gmail.com; Tel.: +966-5444-8025

**Keywords:** toxic, elements, medicinal, plants, ICP-MS, washing, human, health

## Abstract

Local medicinal plants from Madina, Saudi Arabia, are used to cure various diseases. However, some can cause adverse health effects. Five different medicinal plants were collected in the city of Madina: mahareeb (*Cymbopogon*), sheeh (*Artemisia*), harjal (*Cynanchum argel delile*), nabipoot (*Equisetum*), and kafmariam (*Vitex agnus-castus*). In total, four toxic elements including Al, Pb, As, and Cd were analyzed using inductively coupled plasma mass spectrometry (ICP-MS). The range of recoveries fell between 86.1 and 90.6% for all measured elements. Al levels were the highest of any of the studied elements in all plant samples, with *Cymbopogon* showing the highest levels. The range of concentrations of Al was 156–1609 mg/kg. Cd appeared at the lowest levels in all plants samples, with *Vitex agnus-castus* containing this element at the highest levels. Cd concentrations were in the range of 0.01–0.10 mg/kg. A washing process lowered the toxic elements in all plants; average % recoveries were Al (47.32%), As (59.1%), Cd (62.03%), and Pb (32.40%). The calculated human health risk assessment in one dose for toxic elements in all plants was as follows: Al (1.33 × 10^−3^–5.57 × 10^−2^ mg/kg.bw), Pb (0–8.86 × 10^−5^ mg/kg.bw), As (3.43 × 10^−7^–1.33 × 10^−5^ mg/kg.bw), and Cd (0–3.14 × 10^−6^ mg/kg.bw). Medicinal plants are a source of exposure to toxic elements. However, none of the plants in this study exceeded the daily guideline set by the WHO for any element based on conventional use by the local population. We may cautiously conclude that these medicinal plants pose no risk to users based on conventional use.

## 1. Introduction

The use of medicinal plants is an old practice dating back to ancient times. They are used worldwide and very common in many countries. More than 70% of the global population uses medicinal plants to cure different diseases. However, medicinal plants can be a source of exposure to toxic elements depending on their origin and nature [1]. The preparation and use of medicinal plants can be harmful to human health. This has encouraged many researchers to investigate medicinal plants. Several medicinal plants have been investigated, some of which originate from Saudi Arabia such as beckham (*Commiphora opobalsamum*), sweet basil (*Ocimum basilicum*), and juniper (*Juniperus communis*). The study concluded that there were no excess quantities of toxic elements in the investigated plants [2]. A previous study [3] determined levels of toxic and essential elements in some medicinal plants. The medicinal plants included cloves (*Syzygiumaromaticum*), ginger (*Zingiber officinale*), and turmeric (*Curcuma longa*). The study found that daily consumption of 10 g of spices did not expose consumers to any harmful effects. In a recent study [4], the levels of essential and toxic elements were measured in six medicinal plants from China that relieved heat fever and toxicity. The study concluded that the investigated plants (*Viola*, *Andrographis paniculata*, *Rhizoma Menispermi*, *Rhizoma Smilacis Glabrae*, *Prunella*, and *Forsythia*) can be either major or minor sources of the studied elements but are not harmful to human health. Other studies were carried out to determine the levels of various elements in known herbs in Saudi Arabia [2,5]. The study showed that the levels of toxic elements (cadmium and lead) were lower than the values recommended by the World Health Organization (WHO). Calcium and magnesium were found at high levels in some of the investigated medicinal plants (chamomile (*Matricaria chamomile*) and beckham (*Commiphora opobalsamum*)). Some of these herbs tested were cinnamon (*Cinnamomum* sp.), ginger (*Zingiber officinale*), and fenugreek (*Trigonella foenum-graecum*).

Different elements (P, Fe, Cu, K, Mg, B, Al, B, Ca, Fe, and Mn) were measured in plant stimulants and their infusions collected from a market in Czech Republic [6]. Except for Fe and Ca, coffee, compared to tea, is a poor source of these elements. High levels of most of the measured elements were reported in mate (*Ilex paraguariensis A.St.-Hil*.), while rooibos and honeybush had high Ca but showed low levels of the other elements. A previous study [7] explored the utility of the leaves and fruits of medicinal plants, including boar (*Zizyphus jujuba*) and jambhul (*Eugenia jambolana*), in diabetes treatments, and the presence of elements such as Hg, Ni, Cr, Pb, and Cd was investigated. The study concluded that high levels of the various investigated elements in the studied plants were due to environmental pollution. Different elements (Na, Mg, Cu, Zn, Pb, Cd, Fe, and Mn) were measured in various medicinal plants used in Indian curries [8]. The study concluded that the investigated plants such as pudina (*Mentha spicata* L.), pulicha keerai (*Hibiscus cannabinus* L.), and siru keerai (*Amaranthus polygonoides* L.) were rich in essential elements that are beneficial to human health. Antidiabetic medicinal plants (*Catha edulis*, *Erythrina abyssinica*, *Strychnos henningsii Aspilia pluriseta*, and *Bidens pilosa*) were investigated, and levels of different elements (Pb, Cr, Ni, Zn, Cu, Sr, Fe, Mo, and Mn) were measured. These plants can be considered another source of required elements for diabetic patients [9].

Some relevant information was obtained on the usage of the medicinal plants investigated in this study from personal communications with retailers and users. The mahareeb (*Cymbopogon*) is used as a diuretic and to mitigate stomach cramps, sheeh (*Artemisia*) is used to treat diseases related to respiratory and reproductive systems, harjal (*Cynanchum argel delile*) is used to alleviate gastrointestinal pain, and nabi booti (*Equisetum*) and cafmariam (*Vitex agnus-castus*) are used to ease child birth and regulate menstruation, respectively.

In all recent studies, there has been no research carried out on the medicinal plants investigated in this article. Therefore, it is important to investigate local medicinal plants from Madina Al Munawara in Saudi Arabia. The aim of this study was to assess toxic elements in some local medicinal plants from Madina Al Munawara using ICP-MS. Another purpose was to determine if these plants are a source of exposure to toxic elements. A secondary aim was to investigate the effectiveness of washing in the removal and/or extraction of toxic elements from plants. The other aim of this study was a human health risk assessment of ingestion of these medical plants.

## 2. Materials and Methods

### 2.1. Sample Collection and Preparation

Five medicinal plants were purchased from retailers in Madina Al Munawara in the KSA. Mahareeb (*Cymbopogon*), sheeh (*Artemisia*), harjal (*Cynanchum argel delile*), nabipoot (*Equisetum*), and cafmariam (*Vitex agnus-castus*). These medicinal plants are used to cure various diseases. The voucher specimen numbers for these plants are as follows: Cymbopogon (Du Plessis 129,10.-KWAZULU/NATAL.-2930) [10], Artemisia (number 14-12-AS-02 of the identified species were deposited with the Herbarium (BNU) [11], Cynanchum argel delile (Aziz sn. (MO)/Boulos and water) 19142RBGK15763, K)-Egypt [12], Equisetum (W.C. Holmes, J.R. Singhurst and J.N. Mink 15094A) [13], and Vitex agnus-castus (Jundishapur University-Iran; A14311001P) [14].

The families and species for these medicinal plants are as follows: mahareeb: Family: Poaceae, Species: *C. winterianus*; sheeh: Family: Ortheziidae, Species: *P. artemisiae*; harjal: Family: Apocynaceaee, Species: *C. argel*; nabipoot: Order: Equisetales, Species: *E. telmateia*; cafmariam: Family: Labiatae, Species: *V. agnus-castus* [15]. The samples were divided in two portions for each plant. One portion was rinsed with deionized water (washed for about 1 min) and dried at room temperature. The second portion of all samples was washed with deionized water in an ultrasonic bath for ~10 minutes and dried at room temperature. 

### 2.2. Medicinal Plant Digestion

Washed and unwashed portions of the medicinal plants were digested using a previous method [16]. About 0.5 g from each sample (washed and unwashed from each plant) was used for the digestion method. A 4.0 mL portion of nitric acid (69.0% HNO_3_) and 2.0 mL of hydrogen peroxide (30% H_2_O_2_) were used for the digestion process. An Anton-Paar Multiwave microwave (3000 Microwave Sample Preparation System, Graz, Austria) was used for 40 min at a maximum temperature of 125 °C and a total pressure of 20 bar. The digested solution was made up to 10 mL then diluted to 10-fold with deionized water for the analysis.

### 2.3. Elemental Measurement by Using ICP-MS

Concentrations of four elements (Al, Pb, As, and Cd) in the digested sample solutions (*n* = 10) were measured by ICP-MS (iCAP Q, Thermo Fisher Scientific, Waltham, MA, USA). Samples were analyzed in duplicate washed and unwashed portions. iCAPQ ICP-MS Thermo Scientific operating conditions are as follows: power = 1550 W; cool gas flow = 14.1 L/min; nebulizer gas flow = 0.94 L/min; auxiliary gas flow = 0.79 L/min; dwell time = 0.01 s; peristaltic pump speed = 40 rpm; total time for each sample measurement = 3 min.

### 2.4. Chemicals, Reagents, and Analytical Method

Deionized water with high purity; 18 Ω·cm^−1^ was used in all steps of samples preparation and measurements. A single-stock solution was prepared from a mixture of 29 elements at a concentration of 10.0 ± 0.05 μg/mL from ULTRA Scientific (USA). A stock solution (1000 μg/mL) of an internal standard (Sc) was also obtained from ULTRA Scientific (North Kingstown, RI, USA). Fresh standards for analysis were prepared daily from stock solutions in 1% HNO_3_. A concentration of 100 μg/L of Sc was used as an internal standard for the analysis. The calibration standards were used and included the four elements at concentrations in the range from 0 to 40 μg/L.

### 2.5. Quality Control

The performance of the ICP-MS was checked daily prior to analysis. A tune solution (B iCAP) containing U, In, Li, and Co, for the sensitivity and background signal in standard mode, which contained 1 μg/L for each element in 2.0% HNO_3_ and 0.5% HCl. Qtegra software (Thermo Fisher Scientific, Waltham, MA, USA), was used for limits of detection (LODs) related to the four measured elements from the calibration curve of each elements. The LODs (μg/L) were as follows: Al (5.387), Pb (0.080), As (0.008), and Cd (0.01). A continuing calibration verification (CCV) was used for quality control (QC) test for each run. It was performed by measuring 20 μg/L of a mixed standard of all measured elements after each set of five runs. Recoveries for each element, three times (*n* = 3), in one session after each five runs were as follows: Al (86.37), Pb (90.57%), As (86.10%), and Cd (90.47%).

### 2.6. Quality Assurance

The accuracy of the measurement was performed by measuring RCM Hair NCSDC 73347. The results of the measured elements are similar to those of the certified values in the RCM Hair. All elements with certified values (μg/L) and measured values (μg/L) were as follows: Pb (8.8 ± 1.1; 10.49 ± 0.24), As (0.28 ± 0.05; 0.18 ± 0.00), and Cd (0.11 ± 0.03; 0.10 ± 0.00).

### 2.7. Human Risk Assessment: Calculation of the Intake Dose and the Equations

Based on personal communication with users and retailers, the used quantity in one dose for each plant was as follows: mahareeb (3.33 g), sheeh (4.49 g), harjal (7.39 g), nabipoot (3.59 g), and cafmariam (4.33 g). The quantities were calculated for each element contained in each dose of the medical plants in this study. The above used doses from each plant were multiplied by the concentration of each measured element, using the following equation:

D(g) × Cel (μg/g)(1)

where D(g) = a quantity of used dose from a plant in grams and Cel = measured concentration (μg/g) of each element in a plant.

Human risk assessment is calculated as in the following equation:

HRa = (C × D)/BW; [(µg/g × g)/kg.bw]/1000 = mg/kg.bw(2)

where HRa = human risk assessment, C = average toxic element concentration (µg/g), D = the average dose intake of the plant (g), and BW = the average body weight (kg).

### 2.8. Statistical Analysis

Statistical analysis was performed on all plants samples using Excel, *t*-Test (Paired Two Sample for Means). The statistical significance is *p* < 0.05 with 95% confidence level among measured elements.

## 3. Results

Concentration levels (mg/kg) of all measured elements are shown in Table 1 for unwashed plants and washed plants. Washed plants showed lower concentrations for all measured elements than unwashed plants, with few exceptions. This indicates that washing had an effect on reducing elements levels in plants. The reduction in element concentrations following the washing step was up to 80% (Table 1). Mahareeb (*Cymbopogon*) showed higher concentrations for all measured toxic elements, except Pb for sheeh, compared with others plants. Al has reported the highest levels, while Pb, As, and Cd were the lowest in all plants. Moreover, washing was effective in extracting some elements in the infusion from most plants. There was a statistically significant (*p* < 0.05) difference among element concentrations between unwashed and washed plants. However, toxic elements with very low quantities were not removed via washing, nor did they show any statistically significant (*p* > 0.05) difference. Al and As were extracted from all plants except As was not extracted from sheeh. Cd was extracted from cafmariam and mahareeb, but was not removed from any other plant. Pb was only removed from harjal and sheeh. This means that all investigated medicinal plants, except sheeh, constitute a source of Al and As, while only two of them constitute sources of Pb (harjal and sheeh) and two of them sources of Cd (cafmariam and mahareeb). The elements listed in decreasing order in all medicinal plants as follows: Al > Pb > As > Cd.

High percentages of toxic elements were removed from all plants, whereas Pb was not removed/extracted from mahareeb, harjal and nabipoot via washing, while Cd was not removed from sheeh, harjal, or nabipoot. Percentages of 73% Al, 75% As, and 17% Cd were removed from mahareeb via washing. Percentages of 13% Al, 0.2% As, and 53% Pb were removed from sheeh via washing. In harjal, 39% As, 8% Pb, and 41% Al were removed; in nabipoot, 36% As, 37% Al, were removed. In cafmariam, 56% As, 68% Pb, 56% Cd, and 84% Al were removed. Cadmium was not removed in sheeh, harjal, or nabipoot due to low levels. The highest removal percentages (49–84%) for all toxic elements compared with other plants were reported in cafmariam. This means that cafmariam is a source of highly toxic elements compared to other plants. The lowest and the highest measured elements in all plants were Cd and Al, respectively.

The calculated human health risk assessment (mg/kg.bw) for unwashed and washed plants for elements in all plants is shown in Table 2. The calculated dosage of exposure, which is the difference between element quantities in unwashed and washed plants, is also included in Table 2. These dosages of exposure were compared with the provisional tolerable daily intake (PTDI) by WHO as shown in Table 3 [17,18]. The exposure to toxic substances can occur through the inhalation of polluted air, soil, drinking water, and contaminated foods [19]. Since the exposure term used in this study is only regarding the ingestion of medical plants, these medicinal plants pose no risk to users based on conventional use. However, comprehensive toxic elements risk assessment needs to be conducted in future studies to evaluate public or patient health risks.

## 4. Discussion

The investigated medicinal plants in this study are a route of exposure to toxic elements As, Cd, Pb, and Al. The accumulation of toxic elements in the human body will lead to adverse health effects. In contrast, essential elements are important for human health for the prevention and control of diseases [20]. 

Arsenic is a toxic metalloid and causes vitamin A deficiency, which is associated with heart disease and night blindness [21]. Symptoms of acute arsenic toxicity appear within 30 min of ingestion. This causes dryness of the mouth, difficulty swallowing, nerve pain, vomiting, nausea, stomach pain, and diarrhea [21]. Prolonged exposure to arsenic causes skin pigmentation, skin cancer, hyperkeratosis of the feet and hands, and liver damage [22,23]. Chronic exposure to cadmium caused renal damage, cancer, and pulmonary fibrosis. Cadmium is classified as carcinogenic, and has been shown to be toxic in low doses due to its role as an antagonist to some essential elements such as zinc, calcium, copper, and iron [24,25]. Exposure to low levels of lead has been shown to have adverse health effects in humans [24]. The toxicity of lead has been linked to bone marrow and kidney damage, neurotoxicity, and gastrointestinal and neurodevelopmental defects. The adverse effects of lead exposure among children manifested as slowed neurobehavioral development and lower intelligence quotients (IQs) [24,25]. The adverse effects of aluminum include neurological disorders including Alzheimer’s disease, as well as cardiovascular diseases and brain damage [26,27].

Previous studies have been conducted to determine essential and toxic elements in medicinal plants. A similar study [4] was conducted on herbal medicine that is different from the medical plants in this study. Trace elements (Cu, Fe, Zn, Mn, Cr, Ni, Pb, Se, As, and Cd) have been found in Chinese herbal drugs used for relieving heat [4]. Those results showed that higher As (0–10.52 μg/g), Pb (0–6.5952 μg/g), and Cd was not detected in all investigated herbs. This showed that medicinal plants with different origins could show different trends as a source of toxic elements. In a recent study [28] investigating the accumulation of trace elements in two medicinal plants (*Azadirachta indica* and *Ocimum sanctum*) in India found that, for both plants, the lowest concentration of Cd was present in all of the tested elements, which is consistent with the trends of our study. This could demonstrate that different herbal plants in different geographic areas absorb elements in similar ratios. Another recent study [29] investigated trace elements in European medicinal plants, and the following trend was reported: Al (31.21–87.43 μg/g) > Pb (0.6–0.8 μg/g) > Cd (0.02–0.17 μg/g). This trend is the same as the trend in our results for all investigated plants. However, their results [29] for Al and Pb were lower than the results of this study, but their results for Cd agreed with our results. It is worth noting that, even when medicinal plants are grown on different continents, their concentrations of trace elements still follow the same trend. Surprisingly, our study showed high levels of Al and Mn and low levels of As and Cd, which is similar to a recent study [16] on smokeless tobacco (ST). This might lead to the conclusion that all plants grown in similar geographic areas absorb elements in similar proportions.

Our study showed that toxic elements can be removed/extracted via washing from medicinal plants. Removal percentages were up to 56% Cd, 68% Pb, and 80% Al in cafmariam, and 75% As in mahareeb. This demonstrates that cafmariam is the source of the highest measured toxic elements compared with other investigated plants. The lower the percentage (%) recovery, the greater the element concentration that was removed by the washing process. Average % recoveries were Al (47.32%), As (59.1%), Cd (62.03%), and Pb (32.40%) in all plants. The lowest % recoveries for all elements, except for As, which was reported in mahareeb, were reported in cafmariam. The highest % recoveries for all elements, except for Cd, which was in mahareeb, were reported in sheeh. This implies that the toxic elements in cafmariam, compared to mahareeb, were more easily removed via washing. However, the calculated human health risk assessment in all plants (Table 2) is far lower than the guide values provided in Table 3.

The range of Pb was 0.67–2.59 μg/g in a previous study [30] among 30 medicinal herbs consumed in Turkey; in our study, this range was 0.00–3.01 μg/g. This shows that our results for Pb concentrations in all studied plants fall within the range of this previous study. The concentration ranges of Cd and Pb in 10 of the most commonly used medicinal plants in Pakistan [31] were 0.59–1.66 and 3.15–10.63 μg/g, respectively. In our study, the ranges of Cd and Pb were 0.01–0.06 and 0.67–2.59 μg/g, respectively. Our ranges for these two elements are thus lower than those of [31]. However, the average concentrations of Cd (0.029 μg/g) and Pb (0.061 μg/g) in the medicinal plant *Cymbopogon proximus* were both lower than the concentrations in our study (Cd (0.06 μg/g) and Pb (1.77 μg/g)). The concentration ranges of Cd and Pb were 0.003–0.187 and 0.003–0.454 μg/g, respectively, in 33 medicinal plants from Sudan [32]. This shows that our results for Cd were within the range of the results in [32], and the Pb concentrations, albeit with slightly higher values, were similar to those in our study.

The street dust was demonstrated as a source of heavy elements in different traditional markets in Korea [33]. This may suggest that the presence of dust in traditional markets has effects on the medicinal herbs sold to the public. Therefore, retailers should be advised to cover medicinal herbs to protect them from external contamination. Moreover, consumers should be advised to wash these herbs before consumption, which showed clear differences in element concentrations between washed and unwashed herbs (Table 2). 

## 5. Conclusions

We conclude from this study that the medicinal plants we studied are a source of toxic elements. Specifically, in all plants studied, As and Cd were shown to have the lowest concentrations, whereas Al was found to have the highest. The investigated medicinal plants may consider as source of exposure to toxic elements. It is worth mentioning that the washing process worked to remove and extract toxic elements from investigated plants, but Pb and Cd were not removed from all of them. For the human health risk assessment, all calculated toxic element measurements were below the guideline of daily intake levels set by the WHO. There was a statistically significant (*p* < 0.05) difference among element concentrations between unwashed and washed plants, with some exceptions. This may lead us to conclude that some plants have uptake/absorption characteristics for certain elements. Al was shown to have the highest concentration in all plants. Based on the calculated magnitude per dosage the investigated medicinal plants in this study might not pose any risk to human health.

## Figures and Tables

**Table 1:** Concentrations (mg/kg) of the 15 elements measured in unwashed medicinal plants (*n* = 3; mean ± SD).

Elements	Mahareeb	Sheeh	Harjal	Nabipoot	Cafmariam
**Unwashed Plants (mg/kg)**					
As	0.24 ± 0.01	0.05 ± 0.00	0.34 ± 0.01	0.33 ± 0.70	0.103 ± 0.00
Cd	0.06 ± 0.00	0.033 ± 0.00	0.01 ± 0.00	0.04 ± 1.31	0.100 ± 0.00
Pb	1.77 ± 0.02	2.59 ± 0.05	0.67 ± 0.01	0.65 ± 1.97	1.60 ± 0.03
Al	1609.18 ± 17.59	156.40 ± 2.3	212.86 ± 1.15	625.55 ± 1.30	673.25 ± 15.89
**Washed Plants (mg/kg)**					
As	0.06 ± 0.00	0.05 ± 0.00	0.22 ± 0.01	0.21 ± 0.00	0.04 ± 0.00
Cd	0.05 ± 0.00	0.04 ± 0.00	0.01 ± 0.00	0.05 ± 0.00	0.04 ± 0.00
Pb	7.20 ± 0.02	1.21 ± 0.01	0.62 ± 0.01	1.47 ± 0.01	0.51 ± 0.01
Al	428.99 ± 3.71	135.73 ± 3.93	125.02 ± 7.34	397.48 ± 7.38	107.32 ± 2.45

**Table 2:** Calculated human health risk assessment (mg/kg.bw) of toxic elements measured in unwashed and washed plants, based on the commonly used quantity (g) of each plant in one dose. Calculated dosage of exposure (magnitude of the difference in element content before and after washing) is also included.

**Unwashed Plants (mg/kg.bw)**					
	**Mahareeb**	**Sheeh**	**Harjal**	**Nabipoot**	**Cafmariam**
Al	7.61 × 10^−2^	1 × 10^−2^	2.25 × 10^−2^	3.21 × 10^−2^	4.17 × 10^−2^
As	1.13 × 10^−5^	3.2 × 10^−5^	3.61 × 10^−5^	1.69 × 10^−5^	5.57 × 10^−6^
Cd	2.84 × 10^−6^	1.27 × 10^−6^	1.06 × 10^−6^	2.00 × 10^−6^	5.57 × 10^−6^
Pb	8.13 × 10^−5^	1.66 × 10^−4^	5.91 × 10^−5^	2.3 × 10^−5^	9.9 × 10^−5^
**Washed Plants (mg/kg.bw)**					
	**Mahareeb**	**Sheeh**	**Harjal**	**Nabipoot**	**Cafmariam**
Al	2.04 × 10^−2^ (26.8%) ^%R^	8.7 × 10^−3^ (86.8%) ^%R^	1.32 × 10^−2^ (58.7%) ^%R^	2.04 × 10^−2^ (63.5%) ^%R^	3.54 × 10^−4^ (0.8%) ^%R^
As	2.71 × 10^−6^ (24.1%) ^%R^	2.86 × 10^−6^ (89.3%) ^%R^	2.29 × 10^−5^ (63.2%) ^%R^	1.14 × 10^−5^ (67.6%) ^%R^	2.86 × 10^−6^ (51.3%) ^%R^
Cd	2.43 × 10^−6^ (85.4%) ^%R^	2.43 × 10^−6^ (nr)	1.86 × 10^−6^ (nr)	1.06 × 10^−5^ (57.1%) ^%R^	2.43 × 10^−6^ (43.6%) ^%R^
Pb	3.41 × 10^−4^ (nr)	7.71 × 10^−5^ (46.6%) ^%R^	6.57 × 10^−5^ (nr)	6.43 × 10^−5^ (nr)	3.14 × 10^−5^ (18.2%) ^%R^
**Calculated Dose (mg/kg.bw) ^#^**					
	**Mahareeb**	**Sheeh**	**Harjal**	**Nabipoot**	**Cafmariam**
Al	5.57 × 10^−2^ (73.2%) ^$^	1.33 × 10^−3^ (13.2%) ^$^	9.27 × 10^−3^ (41.3%) ^$^	1.17 × 10^−2^ (36.5%) ^$^	4.13 × 10^−2^ (99.2%) ^$^
As	8.57 × 10^−6^ (75.9%) ^$^	3.43 × 10^−7^ (10.7%) ^$^	1.33 × 10^−5^ (36.8%) ^$^	5.49 × 10^−6^ (32.4%) ^$^	2.71 × 10^−6^ (48.7%) ^$^
Cd	4.14 × 10^−7^ (14.6%) ^$^	nc *	nc *	8.57 × 10^−7^ (42.9%) ^$^	3.14 × 10^−6^ (56.4%) ^$^
Pb	nc *	8.86 × 10^−5^ (53.4%) ^$^	nc *	nc *	6.76 × 10^−5^ (81.8%) ^$^

^#^ calculated dose exposure was based on lost quantity of an element via washing. nc * = not calculated. ^%R^ percentage recovery after extraction/washing (the remaining % will be ingested by the user). nr = not removed via washing. ^$^ removed percentage of each elements (this represents a magnitude of the difference in element concentration before and after washing).

**Table 3:** Provisional tolerance weekly intake (PTWI), provisional tolerance daily intake (PTDI), and calculated intake quantity per day (μg/day).

Element	PTWI	PTDI	Calculated Quantity ^#^	Reference
Al	2 mg/kg.bw	0.3 mg/kg.bw/day *	21,000 μg/day	[17]
As	3 μg/kg.bw	0.43 μg/kg.bw/day *	30.1 μg/day	[17]
Cd	25 μg/kg.bw/month	0.83 μg/kg bw/day *	58.1 μg/day	[18]
Pb	25 μg/kg.bw	3.6 μg/kg bw/day *	252 μg/day	[17]

* Calculated intake quantity based on PTWI, ^#^ Equivalent to μg/day for a 70 kg person.
